# Haemodynamic left‐ventricular changes during dobutamine stress in patients with atrial septal defect assessed with magnetic resonance imaging‐based pressure–volume loops

**DOI:** 10.1111/cpf.12781

**Published:** 2022-07-26

**Authors:** Pia Sjöberg, Håkan Arheden, Einar Heiberg, Sigurdur Stephensen, Marcus Carlsson

**Affiliations:** ^1^ Department of Clinical Sciences Lund, Skåne University Hospital, Clinical Physiology Lund University Lund Sweden; ^2^ Wallenberg Centre for Molecular Medicine Lund University Lund Sweden

**Keywords:** atrial septal defect, cardiovascular magnetic imaging, congenital heart defect, heart failure, pressure–volume loops

## Abstract

**Background:**

Atrial septal defect (ASD) results in a left‐to‐right shunt causing right‐ventricular (RV) volume overload and decreased cardiac output from the left ventricle. Pressure–volume (PV) loops enable comprehensive assessment of ventricular function and might increase understanding of the pathophysiology of ASD. The aim of this study was to investigate if left‐ventricular (LV) haemodynamic response to stress in patients with ASD differs from controls.

**Material and Methods:**

Patients with ASD (*n* = 18, age 51 ± 18) and healthy controls (*n* = 16, age 35 ± 13) underwent cardiac magnetic resonance (CMR) and brachial cuff pressure measurements at rest and during dobutamine stress. An in‐house, validated method was used to compute PV loops.

**Results:**

Patients had lower stroke work, potential energy and external power at rest than controls (*p* < 0.001; *p* < 0.05; *p* < 0.05). Stroke work and external power increased and potential energy decreased during stress in patients (*p* < 0.05; *p* < 0.0001; *p* < 0.01) and controls (*p* < 0.0001; *p* < 0.001; *p* < 0.01). Contractility and arterial elastance at rest were higher in patients than controls (*p* < 0.01; *p* < 0.01). Contractility increased during stress in both groups (*p* < 0.0001; *p* < 0.001). There was no difference between patients and controls in arterio‐ventricular coupling.

**Conclusion:**

LV haemodynamic response to stress can be assessed using noninvasive PV loops derived from CMR and brachial blood pressure. Patients with ASD had normal LV energy efficiency, in contrast to other patient groups with decreased cardiac output. Data suggest that patients with ASD had an increased inotropic level at rest with high contractility and heart rate but were able to respond with a further increase during stress, albeit to not as high a cardiac output as controls.

## 1 INTRODUCTION

Atrial septal defect (ASD) is the second most common congenital heart defect and is often not diagnosed until adult life (Campbell, 1970; Liu et al., 2019; Pfitzer et al., 2017). The resulting left‐to‐right shunt causes volume overload of the right atrium and right ventricle and patients often experience shortness of breath, palpitations and decreased exercise capacity (Baumgartner et al., 2010). The left ventricle is also affected, with decreased filling at rest and altered septal function—changes which have been suggested as reasons for decreased exercise capacity (Booth et al., 1988; Hiraoka et al., 2021; Stephensen et al., 2018). Although left‐ventricular (LV) stroke volume (SV) at rest increases soon after ASD closure, peak oxygen uptake (VO_2_) takes a longer time to increase and sometimes does not change at all (Brochu et al., 2002; Rhodes et al., 2002; Stephensen et al., 2019; Takaya et al., 2013). Closure of haemodynamically significant defects has been shown to increase survival and give symptom relief in most cases, but not all patients improve (Konstantinides et al., 1995; Nyboe et al., 2018; Thilén et al., 2016). Also, patients with small ASD are considered not haemodynamically significant, have a reduced lifespan, and impaired exercise capacity (Udholm et al., 2019). Thus, there is still debate whether adult asymptomatic patients with ASD need closure and if patients with small ASD should be considered for closure.

Pressure–volume (PV) loops enable a comprehensive assessment of ventricular function and provide variables such as stroke work, ventricular mechanical efficiency, contractility and arterial elastance (Ea) (Suga et al., 1973). Until recently it had not been possible to reliably obtain PV loops without an invasive procedure. We have previously presented a noninvasive method to investigate PV loops that only need time‐resolved ventricular volumes and brachial pressure, making it clinically readily available (Seemann et al., 2019; Sjöberg et al., 2021). The noninvasive PV loop methodology including the use of peripheral pressure was validated against invasive PV loops with catheterization in pigs, (Seeman 2019). The method is based on the estimation of LV pressure as the product of elastance and volume obtained from cardiac magnetic resonance imaging. The method has been used to show that patients with heart failure due to ischaemic heart disease or dilated cardiomyopathy use more energy to eject the same volume of blood as controls.

The aim of this study was to evaluate whether haemodynamic measures of the left ventricle differ between patients with ASD and controls, at rest and during stress with dobutamine. The purpose was to better understand stress‐induced changes in LV pumping pathophysiology in patients with ASD.

## 2 METHODS

### 2.1 Study design

**Figure 1:**
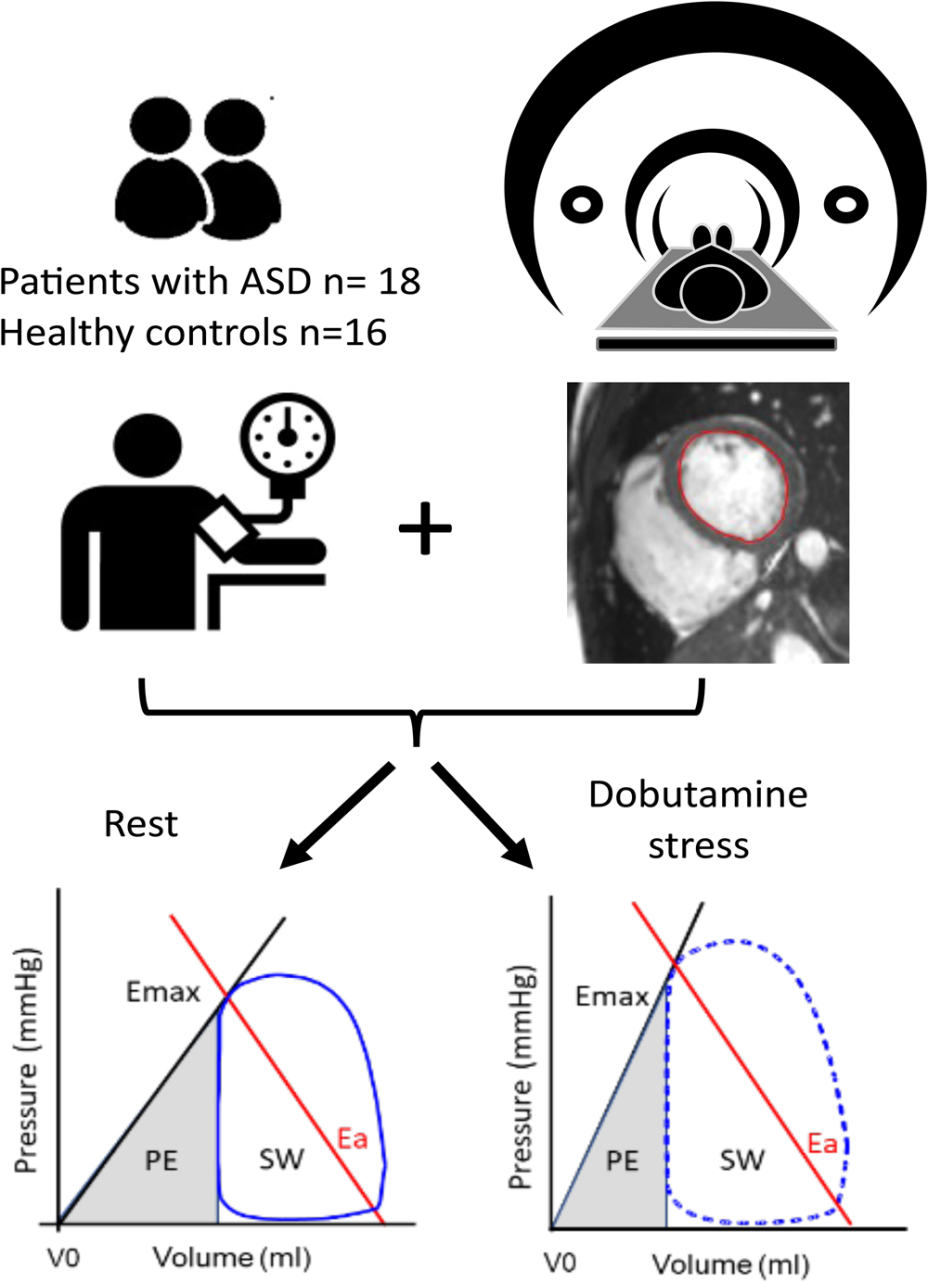
Flow diagram of study design. Patients were examined with cardiac magnetic resonance and brachial pressure at rest and during dobutamine stress. Left‐ventricular endocardial borders were manually delineated in all timeframes, and pressure–volume (PV) loops were derived at rest and during stress. The PV loop area represents stroke work (SW) and the grey triangle corresponds to mechanical potential energy (PE). The slope of the line from maximal ventricular elastance (Emax) and V0 represents the contractility and the negative slope of the red line between the Emax point on the PV loop and the end‐diastolic volume at zero pressure represents the arterial elastance (Ea).

Patients with haemodynamically significant ASD Secundum (Qp/Qs > 1.5), based on clinical findings, echocardiography or cardiac magnetic resonance (CMR) imaging, scheduled for transcatheter closure and in sinus rhythm underwent CMR at rest and with dobutamine stress (Figure 1). In addition, cardiopulmonary exercise testing with continuous gas analysis was performed. Healthy volunteers with normal electrocardiogram (ECG) and blood pressure <140/90 mmHg, no cardiovascular medication, and no medical history of cardiovascular or other systemic disease were examined with the same protocol. Participants’ characteristics are summarized in Table 1.

**Table 1:** Participant characteristics

Mean ± SD	ASD at baseline, *n* = 18	ASD dobutamine, *n* = 16	Controls at baseline, *n* = 16	Controls dobutamine, *n*= 16
Age (years)	51 ± 18**	50 ± 19	35 ± 13	35 ± 13
Sex (male/female)	5/13	4/12	13/3	13/3
BSA (m^2^)	1.9 ± 0.2	1.9 ± 0.2	1.9 ± 0.2	1.9 ± 0.2
Peak VO_2_ (ml/min/kg)	25 ± 6****		46 ± 8	
Peak VO_2_ (% of predicted)	102 ± 15****		133 ± 21	
HR (bpm)	73 ± 11*	123 ± 12*^††††^	65 ± 10	134 ± 12^††††^
HR (% of predicted max)		73 ± 10		73 ± 10
SBP (mmHg)	124 ± 23**	147 ± 21^†††^	122 ± 11	158 ± 22^††††^
DBP (mmHg)	75 ± 16	78 ± 15	74 ± 10	87 ± 10^†††^
LV EDV (ml)	139 ± 32****	126 ± 37**	186 ± 28	162 ± 26^††^
LV ESV (ml)	63 ± 19**	44 ± 17*^††††^	81 ± 17	51 ± 13^††††^
LVSV (ml)	77 ± 18****	82 ± 25***	104 ± 16	110 ± 17^††^
LV EF (%)	55 ± 7	65 ± 8^††††^	56 ± 5	68 ± 4^††††^
LV CO (L/min)	5.5 ± 1.4**	9.8 ± 2.7****^††††^	7.0 ± 1.6	14.0 ± 1.6^††††^
LV CI (L/min/m^2^)	2.9 ± 0.5**	5.3 ± 1.1****^††††^	3.6 ± 0.8	7.2 ± 0.9^††††^
RV EDV (ml)	323 ± 101***	238 ± 91*^††††^	212 ± 44	179 ± 33^†††^
RV ESV (ml)	160 ± 59***	95 ± 51**^††††^	97 ± 28	55 ± 12^††††^
RV SV (ml)	162 ± 50**	143 ± 56	115 ± 20	124 ± 25
RV EF (%)	51 ± 6*	61 ± 11**^†††^	56 ± 6	70 ± 4^††††^
RV CO (L/min)	11.7 ± 3.6***	17.5 ± 6.8^†††^	7.7 ± 1.6	15.7 ± 2.5^††††^
RV CI (L/min/m^2^)	6.4 ± 2.2***	9.7 ± 3.3^††††^	4.0 ± 0.8	8.5 ± 1.5^††††^
Qp/Qs	1.9 ± 0.7	1.5 ± 0.5^††^	‐	‐

*Note*: *p < 0.05, **p < 0.01, ***p < 0.001, ****p < 0.0001 ASD at rest versus controls at rest or ASD at stress vs controls at stress.

^†^ *p *< 0.05, ^††^ *p* < 0.01, ^†††^ *p* < 0.001, ^††††^ *p *< 0.0001 ASD or controls at rest versus stress.

Abbreviations: ASD, atrial septal defect; BSA, body surface area; CI, cardiac index; CO, cardiac output; DBP, diastolic blood pressure; EDV, end‐diastolic volume; EF, ejection fraction; ESV, end‐systolic volume; HR, heart rate; LV, left‐ventricular; peak VO_2_, peak oxygen uptake; Qp/Qs, pulmonary flow/systemic flow; RV, right‐ventricular; SBP, systolic blood pressure; SD, standard deviation; SV, stroke volume.

Participants gave their informed consent before their examinations, and the local ethical review board approved the studies. The principles of the Helsinki declaration were followed.

### 2.2 Cardiac magnetic resonance imaging

Cardiac magnetic resonance imaging with retrospective ECG gating was performed in the supine position using a 1.5 T Achieva (Philips Healthcare). Short‐axis balanced steady‐state free‐precession (bSSFP) cine images covering the entire heart were acquired with retrospective ECG gating. Typical imaging parameters were: acquired temporal resolution 45 ms reconstructed to 30‐time phases per heartbeat; TE/TR 1.4/3 ms; flip angle 60°, slice thickness 8 mm with no gap. Two‐dimensional free‐breathing, through‐plane phase‐contrast (PC) flow measurements were performed in the ascending aorta and pulmonary artery. After examination at rest participants received dobutamine intravenously at 10 µg/kg/min for 3 min, thereafter increased to 20 µg/kg/min. To reach 70% of the predicted maximal heart rate, defined as 220 minus the subject's age, 0.25–0.75 mg of atropine was added if needed after 3 min with dobutamine of 20 µg/kg/min. When the target heart rate was reached, the same images as at rest were acquired. The dobutamine infusion was ongoing during the acquisition of MR images and the heart rate kept above 70% of the predicted maximal heart rate. Blood pressure and heart rate were measured regularly during the examination to assure a steady stress level.

### 2.3 Image analysis

Left‐ventricular endocardial borders were manually delineated in all timeframes. The Segment software (http://segment.heiberg.se) was used, with an in‐house method for the analysis of PV loops (Seemann et al., 2019; Sjöberg et al., 2021). The method estimates of left ventricular pressure using time‐varying elastance and ventricular volume from CMR. The time‐varying elastance is the change in myocardial stiffness during a heartbeat and the peak of the elastance curve, when the ventricular elastance is maximal, corresponds in time just before the minimal left ventricular systolic volume. Left ventricular systolic pressure is approximated from the brachial pressure as (2 × systolic blood pressure)/3 + diastolic blood pressure/3 (Kelly et al., 1992).

### 2.4 Haemodynamic variables

The energy produced by the left ventricle to eject the SV is called stroke work and can be derived from the area within the PV loop, lower panel Figure 1. The mean external power that the left ventricle delivers is calculated as stroke work × (heart rate/60). The potential energy is obtained from the triangular area under the end‐systolic PV relations curve to the left of the PV loop (Suga, 1990). The potential energy is the energy stored in the ventricle at the end of systole and will be converted into heat, e.g., wasted energy. Total ventricular energy consumption of a heartbeat is thus stroke work + potential energy, which has been shown to be proportional to oxygen consumption (Suga, 1979). How much energy is used per ejected volume is calculated as (stroke work + potential energy)/SV. Stroke work as a fraction of total energy consumption, stroke work/(stroke work + potential energy), is a measure of mechanical ventricular efficiency. Myocardial contractility (commonly named Ees for end‐systolic elastance) has been defined as the slope of the relationship of end‐systolic volume to end‐systolic pressure which intersects the *x*‐axis at V_0_ (Westerhof et al., 2010). In the method used in this study, contractility was defined as the slope between the point of maximal ventricular elastance (Emax) and V_0_, which was fixed to 0 (Seemann et al., 2019; Sjöberg et al., 2021). The arterial elastance is represented by the negative slope of the line between the point of Emax on the PV loop and the end‐diastolic volume (Sunagawa et al., 1985).

### 2.5 Exercise performance

Patients performed a maximal exercise test with continuous gas analysis (Carefusion, Oxycon Pro; Jaeger) on a cycle ergometer (939 E, Monark) before the magnetic resonance imaging (MRI) or the day after. The protocol was individualized with incremental increases in workload until exhaustion, at which point peak oxygen uptake (VO_2_) was registered. To ensure maximal exertion, patients were encouraged to continue until the respiratory exchange ratio was 1.1 or more. Twelve‐lead ECG was recorded continuously, and blood pressure was measured manually using a Doppler at rest and every minute during exercise. Peak VO_2_ was presented as ml/min/kg, and as a percentage of the predicted value. Reference values for peak VO_2_ were obtained from the Study of Health in West Pomerania (SHIP) (Gläser et al., 2010).

### 2.6 Statistical analysis

Statistical analysis was performed using GraphPad (v9.1.2). Continuous variables are presented as mean ± standard deviation or median [IQR] according to normal distributions and categorical variables as absolute numbers and percentages. Student's *t*‐test was used to evaluate differences between patients with ASD and healthy volunteers. Paired *t*‐tests were used to assess differences in patients before and after stress. Results with a *p *value of 0.05 or lower were considered statistically significant.

## 3 RESULTS

Characteristics and cardiac volumes of patients and controls are shown in Table 1. Patients were older than controls (*p* < 0.01) and had normal predicted oxygen uptake compared to reference values (Gläser et al., 2010), but lower than the healthy volunteers in this study (*p* < 0.0001). Mean ASD diameter was 15.3 ± 1.3 mm and mean cross‐sectional area indexed to body surface area was 1.0 + 0.2 cm^2^/m^2^. Patients had lower LV end‐diastolic volume (*p* < 0.0001) and end‐systolic volume (*p* < 0.01) at rest than controls. There was a correlation between age and Ea and Ea/Emax in controls (*r* = 0.63, *p* < 0.01 and *r* = 0.53, *p* = 0.034), but not in patients (*r* = 0.46, *p* = 0.055 and *r* = 0.38, *p* = 0.12) (Supporting Information: Figure S1 and Table S1). There was a correlation between age and energy per ejected volume in ASD patients (*r* = 0.54, *p* = 0.02) but not in controls (*r* = 0.28, *p* = 0.29). No other parameters correlated with age in either ASD patients or controls.

Potential energy correlated with peak VO_2_ in patients (*r* = 0.48, *p* = 0.046) but not in controls (*r* = 0.13, *p* = 0.64). Peak VO_2_ did not correlate with any other PV‐loop derived parameters either in ASD patients or controls (Supporting Information: Figure S2).

The ASD size correlated with stroke work (*r* = −0.53, *p* = 0.03), but no correlations with other PV‐loops derived parameters were seen (Supporting Information: Figure S3).

### 3.1 Response to dobutamine stress

Two patients did not complete the dobutamine stress examination due to technical reasons. Volumetric measures, heart rate and blood pressure responses are shown in Table 1. Mean heart rate variation during the dobutamine stress was ±3 bpm for both ASD patients and controls. The left‐to‐right shunt decreased in patients during stress. Patients did not increase cardiac output and heart rate as much as controls in relation to resting values (Figure 3a).

**Figure 2:**
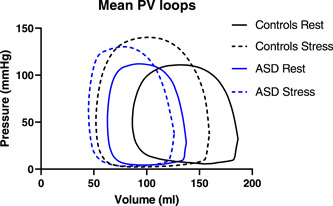
Mean pressure–volume loops for controls (black) and patients with atrial septal defect (ASD) (blue) at rest (solid line) and stress (broken line).

**Figure 3:**
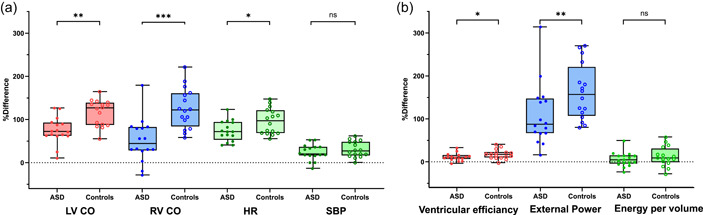
Graphs showing differences in stress response in the left (LV) and right ventricle (RV) between patients with atrial septal defect (ASD) and controls relative to resting values. Box and whiskers show median, IQR, and min to max. Patients are marked with filled circles and controls with open circles. (a) Patients did not increase cardiac output (CO) and heart rate (HR) as much as controls but there was no difference in systolic blood pressure (SBP). (b) Left‐ventricular efficiency and external power did not increase as much in patients with ASD as in controls. Energy per ejected volume remained unchanged in both groups.

The LV PV loops shifted to the left at stress and there was an increase in stroke work, LV efficiency, external power, contractility and Ea/Emax, while potential energy decreased in both patients and controls (Figure 2 and Table 2). External power and LV efficiency did however not increase as much in patients with ASD as in controls (Figure 3b). Arterial elastance and energy per ejected volume remained unchanged in both groups.

**Table 2:** Haemodynamic variables

Mean ± SD	ASD at rest, *n* = 18	ASD at stress, *n* = 16	Controls, *n* = 16	Controls at stress, *n* = 16
Stroke work (J)	0.9 ± 0.3***	1.2 ± 0.4***^†^	1.3 ± 0.2	1.7 ± 0.4^††††^
Potential energy (J)	0.4 ± 0.2*	0.3 ± 0.2^††^	0.5 ± 0.1	0.4 ± 0.2^††^
Stroke work + potential energy (J)	1.4 ± 0.4***	1.4 ± 0.7***	1.8 ± 0.4	2.1 ± 0.5^††^
Ventricular efficiency (%)	69 ± 8	77 ± 9^††††^	70 ± 4	82 ± 4^††††^
External power (J/s)	1.2 ± 0.4*	2.3 ± 0.8****^††††^	1.5 ± 0.4	3.7 ± 0.6^††††^
Contractility, Emax (mmHg/ml)	1.6 ± 0.6**	2.6 ± 1.1*^††††^	1.1 ± 0.2	2.0 ± 0.5^††††^
Arterial elastance, Ea (mmHg/ml)	1.5 ± 0.6**	1.6 ± 0.7	1.1 ± 0.2	1.1 ± 0.3
Ea/Emax	1.0 ± 0.3	0.6 ± 0.2^††††^	1.0 ± 0.2±	0.6 ± 0.1^††††^
Energy per ejected volume (mJ/ml)	18 ± 4	19 ± 3	17 ± 2	193

*Note*: *p *< 0.05, **p < 0.01, ***p < 0.001, ****p < 0.0001 ASD at rest versus controls at rest or ASD at stress vs controls at stress.

^†^ *p *< 0.05, ^††^ *p *< 0.01, ^†††^ *p *< 0.001, ^††††^ *p *< 0.0001 ASD or controls at rest versus stress.

Abbreviations: ASD, atrial septal defect; SD, standard deviation.

Peak VO_2_ did not correlate with the change from rest to stress regarding any of the PV‐loop derived parameters either in ASD patients or in controls (Supporting Information: Figure S4).

## 4 DISCUSSION

This is the first study using noninvasively derived PV loops from CMR to assess the haemodynamic LV response to stress with dobutamine in patients with ASD and healthy volunteers.

Patients with ASD had a smaller increase in cardiac output and external power than controls when going from rest to dobutamine stress. Patients had higher LV contractility and arterial elastance at rest, probably due to an increased inotropic drive to keep up cardiac output despite the decreased SV. This resulted in an Ea/Emax ratio, a measure of ventricular–arterial coupling, that was similar for both groups. Patients and controls used the same amount of energy per ejected volume at rest, and it did not change during stress. This contrasts with patients with heart failure due to ischaemic heart disease or dilated cardiomyopathy, suggesting that the cause of decreased cardiac output is different. Namely, low LV filling volume and pressure in patients with ASD and high LV filling pressure in patients with acquired heart disease and LV failure.

### 4.1 Energy consumption

The myocardial oxygen consumption per heartbeat is correlated with the total ventricular energy consumption of the left ventricle (Suga, 1979). Total ventricular energy consumption consists of stroke work (the energy used to eject the blood to the systemic circulation) plus potential energy (the energy stored in the ventricle at end‐systole and converted to heat) (Suga, 1979). Thus, our results indicate that ASD patients have lower LV oxygen consumption per heartbeat than controls at rest. This could be due to ‘un‐loading’ of the left ventricle with lowered filling due to higher compliance in the RV compared to the LV resulting in lower RV filling pressure permitting left‐right shunting of blood across the atrial septum. The decreased LV filling and thus lower SV is compensated by increased heart rate albeit not sufficient to normalize the external power completely. The increase in oxygen consumption (measured as stroke work plus potential energy) with dobutamine stress seen in healthy controls was not found in patients. In addition, analysis of how much energy is used to eject a certain volume can give a better understanding of cardiac function. In this study, the energy cost per volume was comparable in both patients and controls. Consequently, since patients also had lower heart rate during stress, the external power, the amount of energy per second, was also lower in patients than in controls.

This study shows that ASD patients have lower energy consumption in the LV but RV stroke work is probably higher in ASD patients than in controls since the RV stroke volume in patients is double that of controls and the pulmonary blood pressure is unchanged or higher.

It can be difficult to decide whether a patient with an underfilled left ventricle secondary to the atrial shunt has an LV function good enough to handle the additional volume following ASD closure. A focus for further studies could be to address if the energy consumption per ejected volume might be a prognostic marker in these cases.

### 4.2 Contractility

The definition of myocardial contractility has been extensively discussed over the years (Davidson & Giraud, 2012; Muir & Hamlin, 2020). In this study, however, contractility, Emax, was defined as the slope between V_0_ and the point on the PV loop where the elastance is maximal (just before the end‐systolic volume). Thus, Emax depends on the intrinsic contractility of the myocardium but is also influenced by the anatomical remodelling of the ventricle. Thus, the higher Emax at rest in patients with ASD is likely due to the chronic underfilling of the left ventricle and smaller size of the left ventricle as well as a higher inotropic state. The higher Emax seen in both patients and controls during stress is likely attributed solely to increased myocardial contractility due to increased sympathetic tone. The normal response to stress seen in patients with ASD contrasts with patients with heart failure with preserved ejection fraction. These patients also have higher Emax at rest but in contrast to patients with ASD, they have a blunted increase during stress, due to low intrinsic myocardial function (Kawaguchi et al., 2003; Penicka et al., 2010).

### 4.3 Ventricular‐arterial elastance

Arterial elastance was higher in patients than in controls. Ea correlates with age (Chen et al., 1998) and this difference might be partly explained by the age difference between the groups, but might also be explained by a higher sympathetic tone. The Ea/Emax ratio can be used to assess ventricular‐arterial coupling but it has its limitations: for example, an increase in both Ea and Emax as is commonly seen with increasing age results in a normal ratio (Chen et al., 1998; Ikonomidis et al., 2019). Despite the limitations, Ea/Emax has shown potential to be a prognostic marker (Godfrey et al., 2018; Ky et al., 2013). In our study, both patients and controls had a ratio close to 1 at rest, which has been suggested to be optimal (Chantler et al., 2008; Chirinos, 2013). During stress there was a decrease in Ea/Emax ratio in both groups, suggesting that afterload increases less than LV contractility, thus enhancing cardiac output.

## 5 LIMITATIONS

The method used to obtain PV loops requires unobstructed communication between the left ventricle and the brachial artery. In this study, no patients or controls had signs of stenosis, and so this limitation most likely does not influence the results. The method assumes V_0_ to be zero, an approximation that may not be correct, but validations have shown good agreement between model‐derived measures and in vivo measurements, and so this approximation is judged to be acceptable (Seemann et al., 2019). Another estimation is LV end‐diastolic pressure, though within a range of 0–15 mmHg this seems to have little effect on the variables (Seemann et al., 2019). The control group was rather well trained and younger than the ASD patients which could influence the comparisons, however, there were only modest correlations between VO_2_ or age and some of the PV‐loop derived parameters suggesting this most likely would not affect the overall conclusions of the study. There were also differences between the groups in the distribution between men and women and this may potentially influence the results (Hayward et al., 2001; Yeon et al., 2015). However, these gender differences seem to disappear in patients with acquired heart failure (Mitoff et al., 2007). Multiple individual tests were performed to compare the different variables derived from the PV loops which might imply a risk for multiple test errors.

## 6 CONCLUSION

LV haemodynamic response to stress can be assessed using noninvasive PV loops derived from CMR and brachial blood pressure. Energy consumption is lower for patients than for controls both during rest and during stress. Patients have an increased inotropic level which leads to high contractility and heart rate, but they still have the capacity to respond with further increases during stress, albeit not to the same degree that controls have. Patients with ASD have normal LV energy efficiency, in contrast to patients with heart failure due to ischaemic heart disease or dilated cardiomyopathy, indicating different causes of the decreased cardiac output. Further studies might illuminate if PV loop‐derived variables can provide prognostic information in patients with left‐to‐right shunts.

## CONFLICTS OF INTEREST

Dr. Heiberg is the founder of Medviso AB, Lund, Sweden, the company that produces the Segment software. The remaining authors declare no conflict of interest.

## Supporting information

Supporting information.

Supporting information.

Supporting information.

Supporting information.

Supporting information.

Supporting information.

## Data Availability

Patient data cannot be made available due to data privacy concerns. Other data will be made available upon reasonable request.
