# Supplementary material for: Haemodynamic left‐ventricular changes during dobutamine stress in patients with atrial septal defect assessed with magnetic resonance imaging‐based pressure–volume loops
Source: Clin Physiol Funct Imaging. 2022 Jul 26;42(6):422–9. doi: 10.1111/cpf.12781 (PMC9796342; doi:10.1111/cpf.12781)
Supplement: Supplementary file 1 — Supporting information. [file CPF-42-422-s001.pdf]

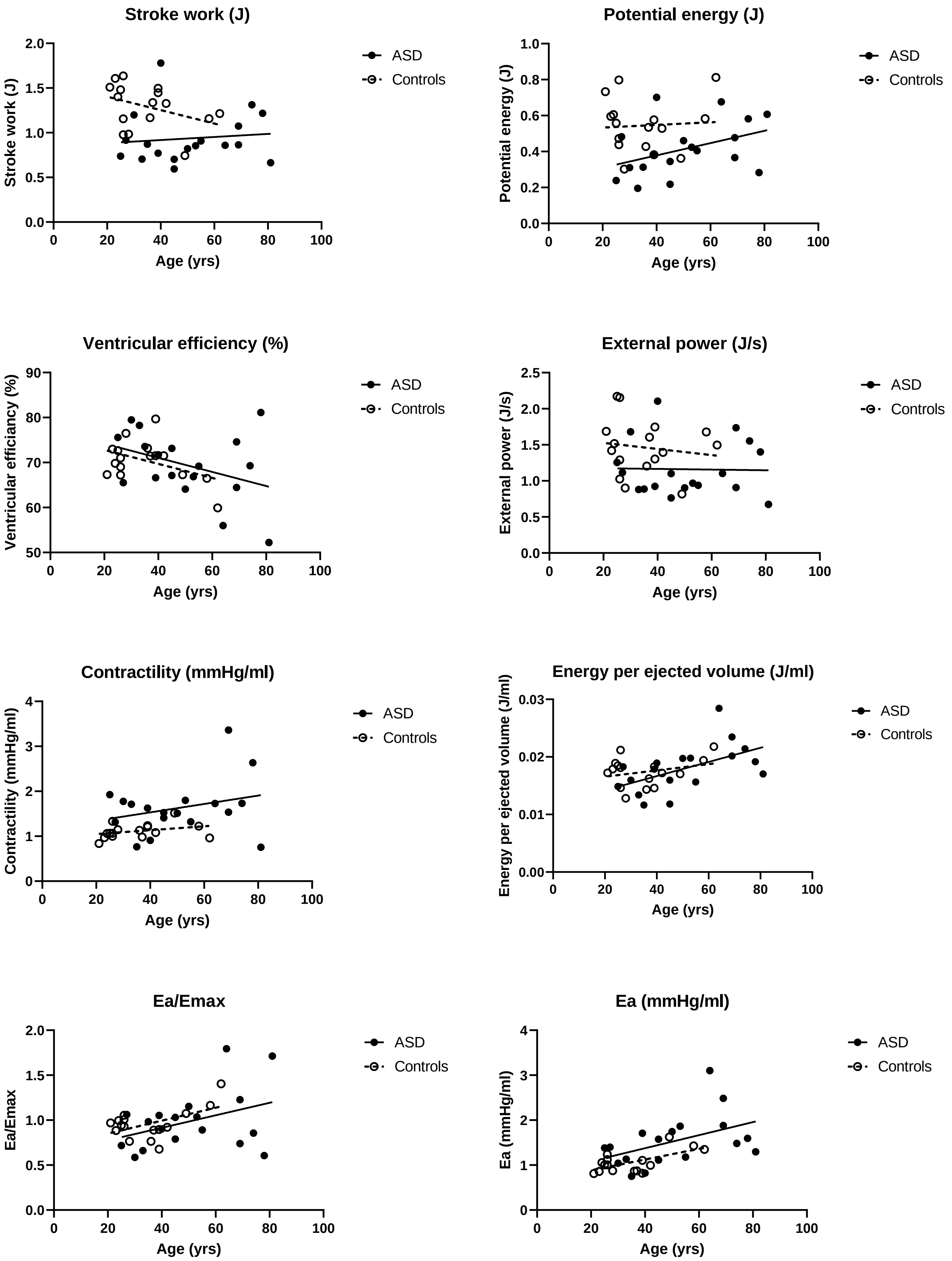

Stroke work (J)
Stroke work (J)
Age (yrs)
ASD
Controls
Potential energy (J)
Potential energy (J)
Age (yrs)
ASD
Controls
Ventricular efficiency (%)
Ventricular efficiancy (%)
Age (yrs)
ASD
Controls
External power (J/s)
External power (J/s)
Age (yrs)
ASD
Controls
Contractility (mmHg/ml)
Contractility (mmHg/ml)
Age (yrs)
ASD
Controls
Energy per ejected volume (J/ml)
Energy per ejected volume (J/ml)
Age (yrs)
ASD
Controls
Ea/Emax
Ea/Emax
Age (yrs)
ASD
Controls
Ea (mmHg/ml)
Ea (mmHg/ml)
Age (yrs)
ASD
Controls
