# Supplementary material for: Haemodynamic left‐ventricular changes during dobutamine stress in patients with atrial septal defect assessed with magnetic resonance imaging‐based pressure–volume loops
Source: Clin Physiol Funct Imaging. 2022 Jul 26;42(6):422–9. doi: 10.1111/cpf.12781 (PMC9796342; doi:10.1111/cpf.12781)
Supplement: Supplementary file 2 — Supporting information. [file CPF-42-422-s002.pdf]

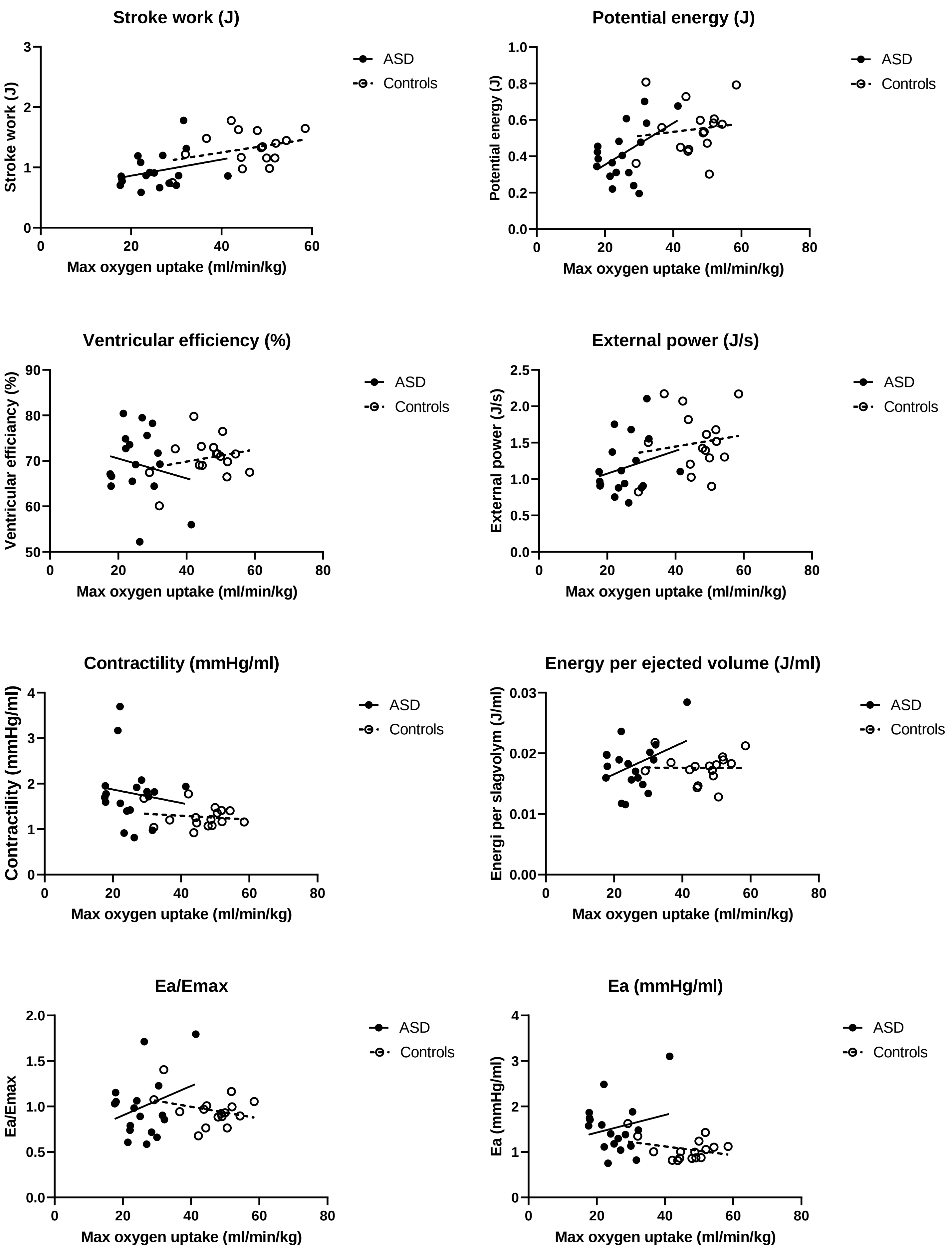

Stroke work (J)
Stroke work (J)
Max oxygen uptake (ml/min/kg)
ASD
Controls
Potential energy (J)
Potential energy (J)
Max oxygen uptake (ml/min/kg)
ASD
Controls
Ventricular efficiency (%)
Ventricular efficiancy (%)
Max oxygen uptake (ml/min/kg)
ASD
Controls
External power (J/s)
External power (J/s)
Max oxygen uptake (ml/min/kg)
ASD
Controls
Contractility (mmHg/ml)
Contractility (mmHg/ml)
Max oxygen uptake (ml/min/kg)
ASD
Controls
Energy per ejected volume (J/ml)
Energi per slagvolym (J/ml)
Max oxygen uptake (ml/min/kg)
ASD
Controls
Ea/Emax
Ea/Emax
Max oxygen uptake (ml/min/kg)
ASD
Controls
Ea (mmHg/ml)
Ea (mmHg/ml)
Max oxygen uptake (ml/min/kg)
ASD
Controls
