# Supplementary material for: Haemodynamic left‐ventricular changes during dobutamine stress in patients with atrial septal defect assessed with magnetic resonance imaging‐based pressure–volume loops
Source: Clin Physiol Funct Imaging. 2022 Jul 26;42(6):422–9. doi: 10.1111/cpf.12781 (PMC9796342; doi:10.1111/cpf.12781)
Supplement: Supplementary file 3 — Supporting information. [file CPF-42-422-s004.pdf]

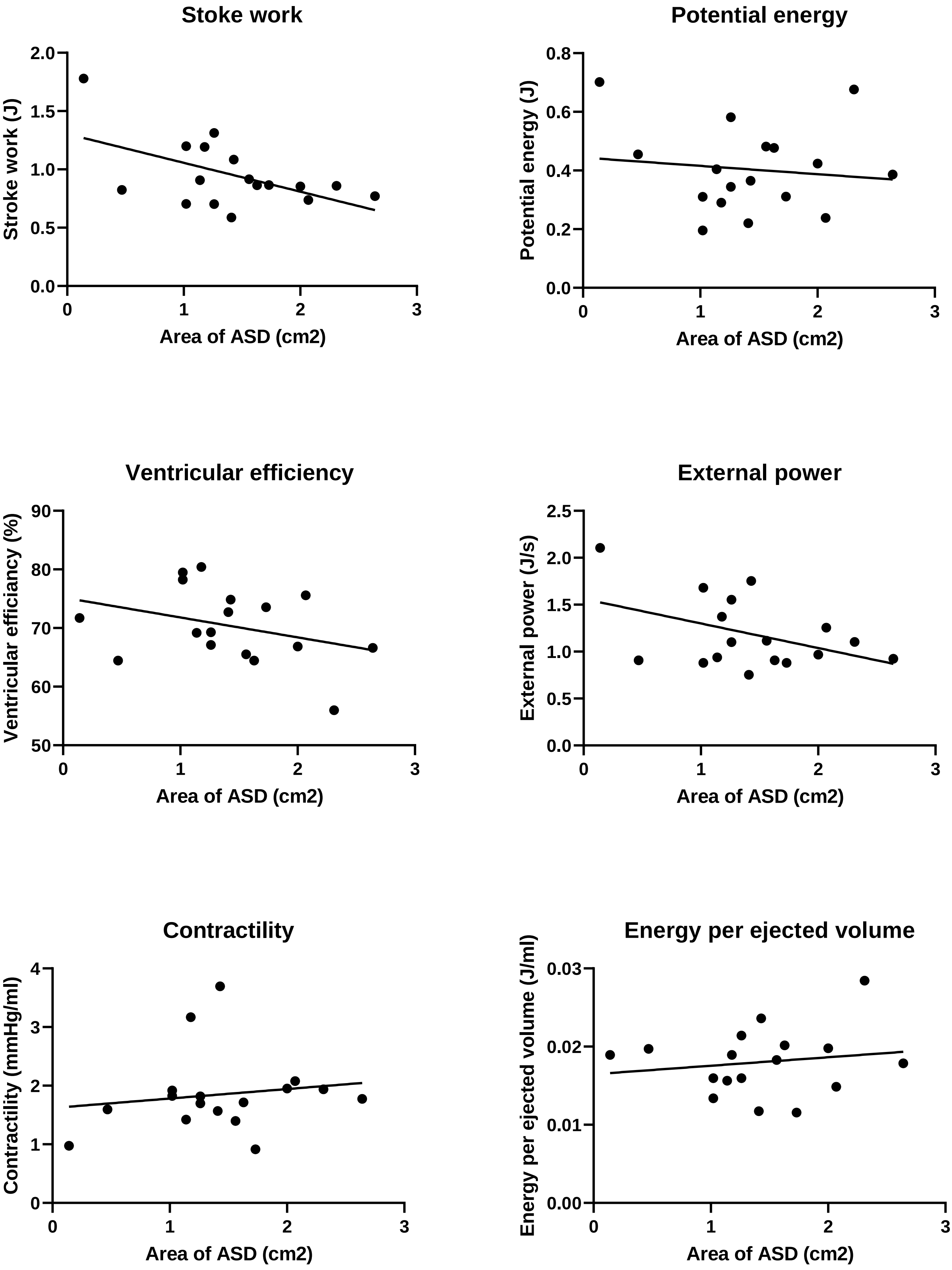
Stoke work
Stroke work (J)
2.0
1.5
1.0
0.5
0.0
0
1
2
3
Area of ASD (cm2)
Potential energy
Potential energy (J)
0.8
0.6
0.4
0.2
0.0
0
1
2
3
Area of ASD (cm2)
Ventricular efficiency
Ventricular efficiancy (%)
90
80
70
60
50
0
1
2
3
Area of ASD (cm2)
External power
External power (J/s)
2.5
2.0
1.5
1.0
0.5
0.0
0
1
2
3
Area of ASD (cm2)
Contractility
Contractility (mmHg/ml)
4
3
2
1
0
0
1
2
3
Area of ASD (cm2)
Energy per ejected volume
Energy per ejected volume (J/ml)
0.03
0.02
0.01
0.00
0
1
2
3
Area of ASD (cm2)
