# Supplementary material for: Haemodynamic left‐ventricular changes during dobutamine stress in patients with atrial septal defect assessed with magnetic resonance imaging‐based pressure–volume loops
Source: Clin Physiol Funct Imaging. 2022 Jul 26;42(6):422–9. doi: 10.1111/cpf.12781 (PMC9796342; doi:10.1111/cpf.12781)

Difference in stroke work rest-stress

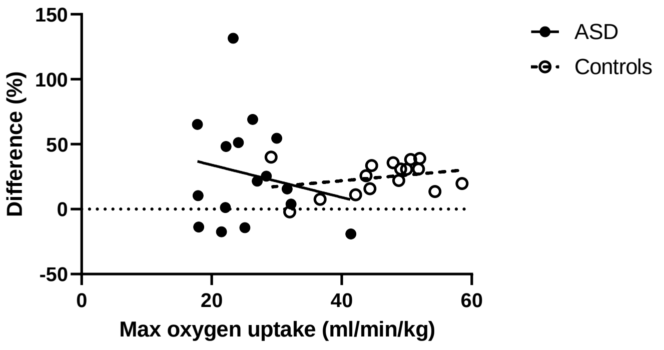

Difference in potential energy rest-stress

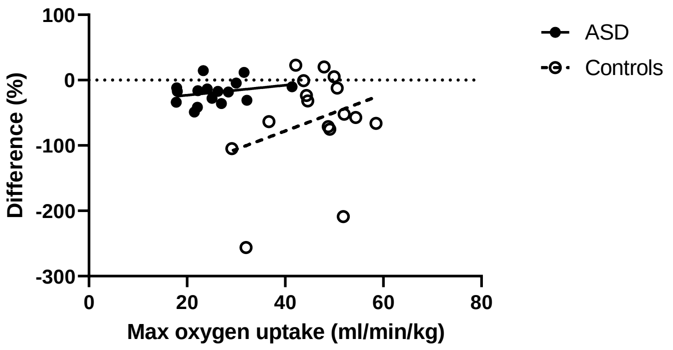

Difference in ventricular efficiency rest-stress

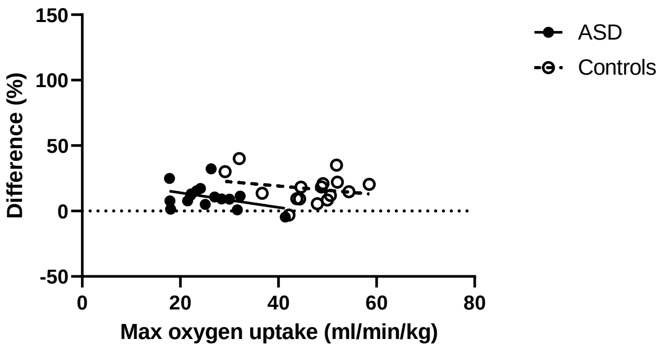

Difference in external power rest-stress

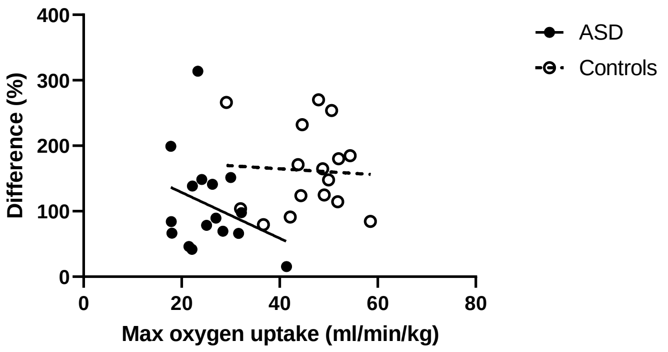

Difference in contractility rest-stress

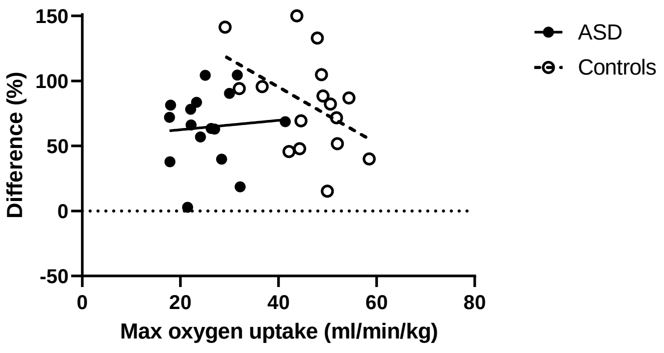

Difference in energy per ejcted volume rest-stress

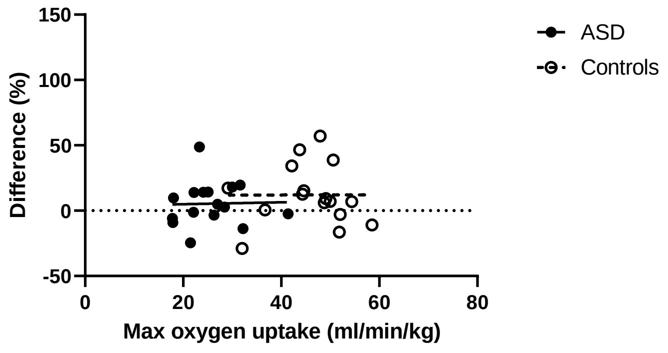

Difference in Ea/Emax rest-stress

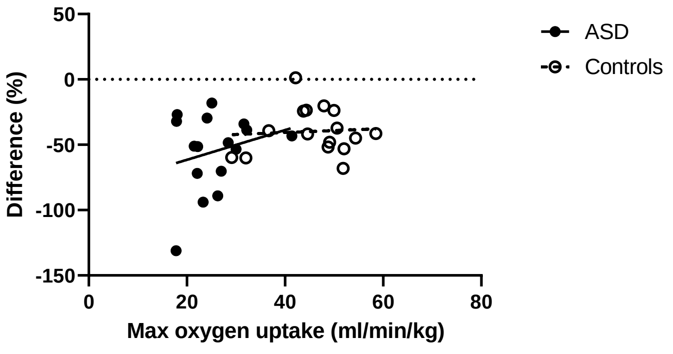

Difference in Ea rest-stress

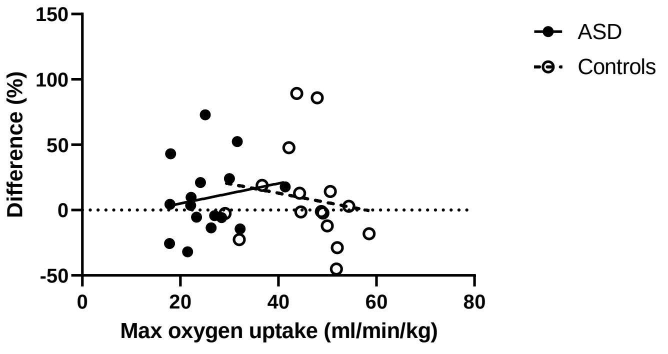

Supplement: Supplementary file 4 — Supporting information. [file CPF-42-422-s006.pdf]
