# Supplementary material for: Haemodynamic left‐ventricular changes during dobutamine stress in patients with atrial septal defect assessed with magnetic resonance imaging‐based pressure–volume loops
Source: Clin Physiol Funct Imaging. 2022 Jul 26;42(6):422–9. doi: 10.1111/cpf.12781 (PMC9796342; doi:10.1111/cpf.12781)
Supplement: Supplementary file 5 — Supporting information. [file CPF-42-422-s003.pdf]

Figure S1. Correlation between age and hemodynamic parameters There was a correlation between age and Ea and Ea/Emax in controls (r=0.63, p<0.01 and r=0.53, p=0.034), but not in patients (r=0.46, p=0.055 and r=0.38, p=0.12). There was a correlation between age and energy per ejected volume in ASD patients (r=0.54, p=0.02) but not in controls (r=0.28, p=0.29). No other parameter correlated with age in either ASD patients (stroke work: r=0.11, p=0.67; potential energy: r=0.41, p=0.091; ventricular efficiency: r=-0.37, p=0.13; external power: r= 0.02, p=0.93; contractility: r= 0.27, p=0.27) or patients (stroke work: r= 0.38, p=0.15; potential energy: r=0.06, p=0.82; ventricular efficiency: r=-0.44, p=0.090; external power: r= 0.14, p=0.61; contractility: r= 0.33, p=0.21)

Figure S2. Correlation between peak VO2 and hemodynamic parameters Potential energy correlated with peak VO2 in patients (r=0.48, p=0.046) but not in controls (r=0.13, p=0.64). Peak VO2 did not correlate with any other PV-loop derived parameters either in patients (stroke work: r=0.29, p=0.24; ventricular efficiency: r=-0.18, p=0.47; external power: r=0.25, p=0.31; contractility: r= 0.13, p=0.60; energy per ejected volume: r=0.39, p=0.11; Ea: r=0.21, p=0.40; Ea/Emax: r=0.30, p=0.22) or in controls (stroke work: r=0.32, p=0.22; ventricular efficiency: r=0.24, p=0.37; external power: r=0.15, p=0.58; contractility: r= 0.14, p=0.60; energy per ejected volume: r= 0.010, p=0.97; Ea: r=-0.32, p=0.22; Ea/Emax: r= 0.30, p=0.26).

Figure S3. Correlation between ASD size and hemodynamic parameters There was a correlation between the ASD size and stroke work (r=-0.53, p=0.03, but no correlations between potential energy (r=-0.12, p=0.65), ventricular efficiency (r=-0.33, p=0.19), external power (r=-0.43, p=0.08), contractility (r=0.15, p=0.57) or energy per ejected volume (r=0.16, p=0.54)

Figure S4. Correlation between peak VO2 and hemodynamic parameters' change from rest to stress Peak VO2 did not correlate with the change from rest to stress regarding any of the PV-loop derived parameters either in ASD patients (stroke work: r=-0.19, p=0.48; potential energy: r=0.27, p=0.31; ventricular efficiency: r=-0.39, p=0.14; external power: r=0.30, p=0.26; contractility: r=0.079, p=0.77; energy per ejected volume: r=0.027, p=0.92; Ea: r=0.17, p=-0.53; Ea/Emax: r=0.23, p=0.37) or in controls (stroke work: r=0.28, p=0.30; potential energy: r=0.29, p=0.27; ventricular efficiency: r=-0.23, p=0.38; external power: r=-0.057, p=0.84; contractility: r= 0.45, p=0.078; energy per ejected volume: r=0.0008, p=1.0; Ea: r=-0.15, p=0.57; Ea/Emax: r=0.066, p=0.81)
