# Supplementary material for: Haemodynamic left‐ventricular changes during dobutamine stress in patients with atrial septal defect assessed with magnetic resonance imaging‐based pressure–volume loops
Source: Clin Physiol Funct Imaging. 2022 Jul 26;42(6):422–9. doi: 10.1111/cpf.12781 (PMC9796342; doi:10.1111/cpf.12781)
Supplement: Supplementary file 6 — Supporting information. [file CPF-42-422-s005.pdf]

Table S1. Younger than 65 years. ASD 42±12 years, Controls 35±13 years, p=0.10

Mean±SD	ASD at rest n=14	ASD at stress n=13	Controls n=16	Controls at stress n=16
Stroke work (J)	0.9±0.3 ***	1.2±0.5 **	1.3±0.2	1.7±0.4 ††††
Potential energy (J)	0.4±0.2 *	0.4±0.2	0.5±0.1	0.4±0.2 ††
Stroke work + Potential energy (J)	1.2±0.5 ***	1.4±0.7 **	1.8±0.4	2.1±0.5 ††
Ventricular efficiency (%)	70±6	77±10 †	70±4	82±4 ††††
External power (J/s)	1.1±0.4 *	2.4±0.8 ***††††	1.5±0.4	3.7±0.6 ††††
Contractility, Emax (mmHg/ml)	1.5±0.3 **	2.6±0.6 *††††	1.1±0.2	2.0±0.5 ††††
Arterial Elastance, Ea (mmHg/ml)	1.4±0.6 *	1.7±0.8 **	1.1±0.2	1.1±0.3
Ea/Emax	1.0±0.3	0.7±0.3 ††	1.0±0.2	0.6±0.1 ††††
Energy per ejected volume (mJ/ml)	17±4	19±4	17±2	19±3

ASD, atrial septal defect

* p<0.05, ** p<0.01, *** p<0.001, **** p<0.0001 ASD at rest vs controls at rest or ASD at stress vs controls at stress

† p<0.05, †† p<0.01, ††† p<0.001, †††† p<0.0001 ASD or controls at rest vs stress
